# Evaluating the effectiveness of the cardiovascular assessment screening program with nurse practitioners and patients: results of a cluster randomised controlled trial

**DOI:** 10.1186/s12875-024-02432-2

**Published:** 2024-05-24

**Authors:** Jill Bruneau, Donna Moralejo, Karen Parsons

**Affiliations:** Faculty of Nursing, Memorial University of Newfoundland, 323 Prince Philip Drive, St. John’s, NL A1B 3X8 Canada

**Keywords:** Randomized controlled trial, Nurse practitioners, Screening, Cardiovascular disease, Primary care

## Abstract

**Background:**

There is inconsistent utilisation of clinical practice guidelines (CPGs) for cardiovascular disease (CVD) screening and management by healthcare professionals to identify CVD risk factors early and to intervene using current recommendations. To address this issue, the Cardiovascular Assessment Screening Program (CASP) was developed, implemented, and evaluated. This manuscript reports on the second phase of an exploratory sequential mixed methods study that tested the effectiveness of the CASP with nurse practitioners (NPs) and patients in Canada.

**Methods:**

A two-armed, non-blinded, cluster randomised controlled trial (cRCT) compared the NP-led implementation of CASP with usual care by NPs in community practice clinics across one Canadian province. The NPs were the cluster variable as their screening practices could be affected by their educational training, resources, or other factors. NPs were eligible for inclusion in the study if they were located in different urban and rural community settings and could conduct follow-up visits with patients. NPs recruited and enrolled the patients from their own practices as participants if they were healthy individuals, aged 40–74 years, with no established CVD or vascular disease. Researchers randomly allocated the NPs (*n* = 10) to the intervention group (IG) or the control group (CG).

**Results:**

Eight (8) NPs and 167 patients participated in the cRCT study. Patient participant-level data were analysed by the originally assigned groups IG (*n* = 68) and CG (*n* = 99). Utilising GLM (generalized linear modeling) more IG patients (90%; *n* = 61) received comprehensive CVD screening compared to the CG patients (2%; *n* = 2), RR = 30.2, 95% CI [8.76, 103.9], *p* < .0001, controlling for the effect of NP and BP category.

**Conclusion:**

NP implementation of CASP was effective for comprehensive screening compared to usual care and led to identifying previously unknown CVD risk factors, calculated FRS, heart health priorities and personalised goal-setting.

**Trial registration:**

ClinicalTrial.gov ID#: NCT03170752, date of registration 2017/05/31.

## Background

Cardiovascular disease (CVD) is the leading cause of death globally and accounts for about 30% of all deaths [1, 2]. CVD morbidity results in lost years of life, reduced productivity, and decreased quality of life for many individuals and families [3]. CVD develops because of a combination of genetic, social, and environmental influences over a number of years with CVD incidence increasing with advancing age [4]. Control of risk factors is therefore critical to the prevention of CVD. Identification of these risk factors and conditions early in the lifespan, through consistent screening, can lead to reductions in CVD morbidity and premature mortality [5]. Different types of health care providers (HCPs) play key roles in the identification and management of CVD risk factors. Our study focused on one group of HCPs, nurse practitioners (NPs), who, like general practitioners, focus on providing primary health care in community settings. NPs are advanced practice nurses who assess, diagnose, order diagnostic tests, and prescribe medications.

In Canada, specific guidelines for CVD screening and management of risk factors have been published, such as the Canadian Cardiovascular Harmonized Guideline Endeavour (C-CHANGE) [6]. The C-CHANGE guideline contains in-depth information regarding CVD screening and management. As well, this guideline is multifaceted addressing many different risk factors and conditions. However, HCPs are often unaware of the current C-CHANGE guideline and inconsistently identify, manage, and document CVD risk factors in daily clinical practice [7]. In fact, HCPs often screen for single CVD risk factors opportunistically during a clinic visit rather than using a comprehensive approach to systematically screen for multiple risk factors simultaneously [8]. Using a systematic comprehensive approach is important to determine the level of CVD risk in early adulthood so to potentially reduce morbidity and premature CVD mortality [9]. In our study, comprehensiveness was defined as screening for 9–10 specified risk factors or risk conditions during the clinic visit or patient encounter.

During phase 1 of a multi-phase exploratory sequential mixed methods study, our research team developed the Cardiovascular Assessment Screening Program (CASP), based on the current C-CHANGE guideline, and focused on comprehensive, systematic, contextually relevant CVD screening and management. The CASP intervention consisted of four different components: (1) a CVD database, (2) a CASP website, (3) a HCPs’ toolkit, and, (4) an online educational module. The components of CASP guided the participating NPs in screening and managing risk factors. For example, the CVD database was set up as a worksheet to guide NPs through CVD screening, specifying what to assess, while also providing a tool for documentation. The other CASP components were also easily accessible online in daily clinical practice through a website, a HCPs’ toolkit, and an educational module. The CASP website, accessible for NPs and patients, contained background information about the research study and tools for accurate measurement to ensure consistent data collection. CVD screening and recommendations for management of the risk factors were based on the C-CHANGE guideline embedded in the CASP website intervention. A toolkit and an online education module also aided the NPs in CVD screening and management. Details of the CASP intervention and its development in the first phase of our mixed methods study are described elsewhere [10, 11].

During phase 2 of the mixed methods study, a cluster randomised controlled trial (cRCT) was chosen to examine the effectiveness of a NP-led implementation of CASP to improve comprehensive CVD screening with patients across one Canadian province, Newfoundland and Labrador (NL). Random assignment of NPs working in different communities across NL occurred. NPs in the intervention group (IG) recruited patients from their own practice and implemented CASP with them. NPs in the control group (CG) similarly recruited their own patients and provided usual care. The main objective of the cRCT was to test the effectiveness of the CASP intervention to determine whether its implementation by NPs with patients in the IG versus NPs providing usual care in the control group (CG) resulted in comprehensive CVD screening of their patients. In this paper, we report on the outcomes of the CASP implementation at the patient participant level taking into account the effect of the NP. The NPs were the cluster level variable. As their usual practice may be affected by their education, available resources, geographic location, etc., it was important to control for the effect of the cluster in the analysis, but the primary outcome of interest was the effect at the level of the individual patient participant. Reporting was guided by the extension of CONSORT 2010 for cluster trials [12].

## Methods

### Trial design

We designed a two-armed, non-blinded cRCT in community settings across NL to compare the implementation of the CASP intervention with care as usual. The study period was from September 2017 to November 2018, with data collection between March and November 2018. In this cRCT, the NPs were from eight different locations or sites across the province of NL. There were no NPs from the same practice sites (e.g., clinics) enrolled in this study. Each NP recruited patients from their own clinical practice sites. We obtained approval from the Health Research Ethics Board (HREB) and the Research Proposal Approval Committees (RPACs) in the regional health authorities (RHAs) in NL, Canada. We addressed key ethical considerations of potential risks, harms, and benefits, informed consent, confidentiality, and cost considerations. There were no changes to the methods after trial commencement; however, we did experience significant challenges in recruitment of the NPs for the study. Details of the recruitment procedures and challenges, along with potential strategies to address recruitment of busy HCPs, are described elsewhere [13]. This investigation conforms to the principles outlined in the Declaration of Helsinki.

### Participants

NPs, recruited by the researchers, were eligible for inclusion if they practiced in community settings in NL, either rural or urban, within RHAs. The NPs had to be able to access eligible patients through their community clinics and follow up with these same patients through regular clinic visits. Patients were recruited by the NPs in their community practice clinics. The NPs identified age-eligible patients who were then given information about the study and a heart health self-assessment form to complete. If patients were interested in learning more or participating in the study, any questions were answered and eligibility was further assessed by the NP. Patients were eligible to participate if they were healthy, asymptomatic individuals between the ages of 40–74 years, but had no established CVD or vascular disease. Researchers wanted to focus this research study on a population with no established CVD or vascular disease. The rationale for selecting this population was because this population is not normally routinely screened and it was important to know what their risk factors were for timely management.

### Interventions

NPs in the IG implemented the CASP intervention through collaboration with their patient participants. Prior to the data collection period, each NP of the IG participated in one webinar outlining the research study and the correct procedures for implementing CASP. Follow-up during data collection with each NP was individualised by the researchers with support provided, as needed, via phone calls, emails, or additional webinars. The CASP intervention contained novel tools, based on the current C-CHANGE guideline and created specifically by researchers for this study.

NPs in the IG completed the intervention, i.e., CVD screening using CASP, over two patient visits, during the data collection period. The CVD database was set up as a guide for NPs to use so that they knew what data to focus on collecting and documented data could be used in later analysis. The first clinic visit consisted of collecting demographic data, patient self-report of risk factors, and physiological measurements, including BP, heart rate, weight, height, body mass index (BMI), and waist circumference (WC), with requisitions given to each patient participant for specific screening bloodwork prior to the next clinic visit. During the subsequent patient visit, NPs interpreted blood work results, calculated the Framingham Risk Score (FRS) to determine CV risk for an adverse event in the future and compared their chronological age with the computed Heart Age, and then communicated these results with their patient participants. Then, NPs and their patients collaborated to set priorities for heart health and personalised heart health goals [14]. Both patient and NP priorities were documented in the CVD database. Any further follow-up between the NPs and patients was not part of the study. NPs in the CG participated in a webinar with researchers about recruitment of patient participants into the cRCT, then provided care as usual to their patients during the study period. Subsequent questions from the CG NPs were answered by researchers via email or phone calls.

### Outcomes

All of the outcomes reported in this paper focused on the participant level analysis while taking into account the effect of the NP. The primary outcome was comprehensiveness of CVD screening, defined as having nine or ten of the following components assessed and documented: (a) patient’s age; (b) family history of premature coronary artery disease (CAD); (c) FRS; (d) smoking status; (e) BMI; (f) WC; (g) blood pressure; (h) lipid profile; (i) A1C; and (j) psychological stress.

The secondary outcomes were the following: (1) the identification of CVD risk factors, (2) level of CVD risk according to the FRS and Heart Age, and (3) the identification of NPs and patients’ priorities for heart health and personalised goal setting. These secondary outcomes were measured by analysing the documentation in the CVD database based on the patient participants’ clinic visits in the IG and by reviewing the patients’ charts in the CG. One researcher reviewed the charts of those patients who had consented to participate, using a chart review form developed by the research team. The following information was extracted from the patients’ charts: demographics, history and physical findings, physiological measurements, laboratory data, and NP recommendations for patient care during clinical visits.

### Sample size

The sample size estimation for this study was determined using the proportion of eligible patients who were comprehensively screened as the outcome measure of interest. A study that considered the effectiveness of a national risk assessment program for patients aged 40–74 years found that approximately 40% had complete Health Checks and 60% had partial Health Checks among high risk patients in the UK National Health Service (NHS) Health Check Program [15]. For our study we assumed that 40% of the screening would be comprehensive in the CG and considered that comprehensive screening of 70% of patients seen by the NPs in the IG would indicate an effective intervention. Using a two-sided alpha of 0.05 and 90% power, the sample size was calculated to be 250 patients (125 patients per group). Assuming that 20% of those approached would refuse, our target was 10 NPs, each recruiting 30 patients, for a total of 300 patients. At the end of the data collection period, there were four NPs in each group, with 9 to 30 patient participants in per NP. The intracluster correlation coefficient (ICC) calculated was 0.82. Researchers accounted for the effect of the NP using generalized linear modeling (GLM) in STATA 17 software [16].

### Randomisation and consent

Using a random number generator in STATA, the Principal Investigator (PI) allocated NPs, one to one, to either the intervention or the control group [17]. In this study, the PI allocated the NPs, not the individual patient participant level, and the effect of the specific NP cluster was controlled for in the analysis. Allocation concealment was at the cluster level such that neither the investigators nor the NPs knew the allocation sequence in advance. Researchers sought and obtained informed consent from all NPs in the IG and CG prior to the randomisation to the groups. Following allocation of the NPs to the intervention or control group, researchers educated the NPs about the recruitment procedures and the patient eligibility criteria. Then, the NPs followed a script to obtain informed written consent from the patient participants and enrolled them into the trial, and were given opportunities to refuse to participate without impacting their care. Eligibility and consent were verified by the researchers.

### Statistical methods

The data were analysed using STATA 17 statistical software [16] and all analyses were by the originally assigned groups. The adjusted relative risk was calculated for the primary outcome using GLM and cluster robust standard errors to control for the effect of the NP. Descriptive statistics were used to compare differences between the intervention and the control group in terms of the identification of patients at risk for CVD, the priorities identified by the patients and the NPs in the IG, and the recommendations made by the NPs in the IG. Differences between patient baseline characteristics were tested using chi square (χ^2^). The adjusted relative risk was calculated for the primary outcome (comprehensive screening or not) using binomial GLM and cluster robust standard errors to control for the effect of the NP, which was the cluster variable. Hierarchical modelling was conducted, with potential variables assessed one at a time before being dropped. Models were compared using the likelihood ratio test. The following were assessed to see if they were predictors or confounders or if they could be dropped from the model: age, gender, BP category, diabetes, smoking status, renal dysfunction, and RHA. The process was repeated until all variables were assessed and the final model established. The assumptions for GLM were assessed as being met.

## Results

**Fig. 1:**
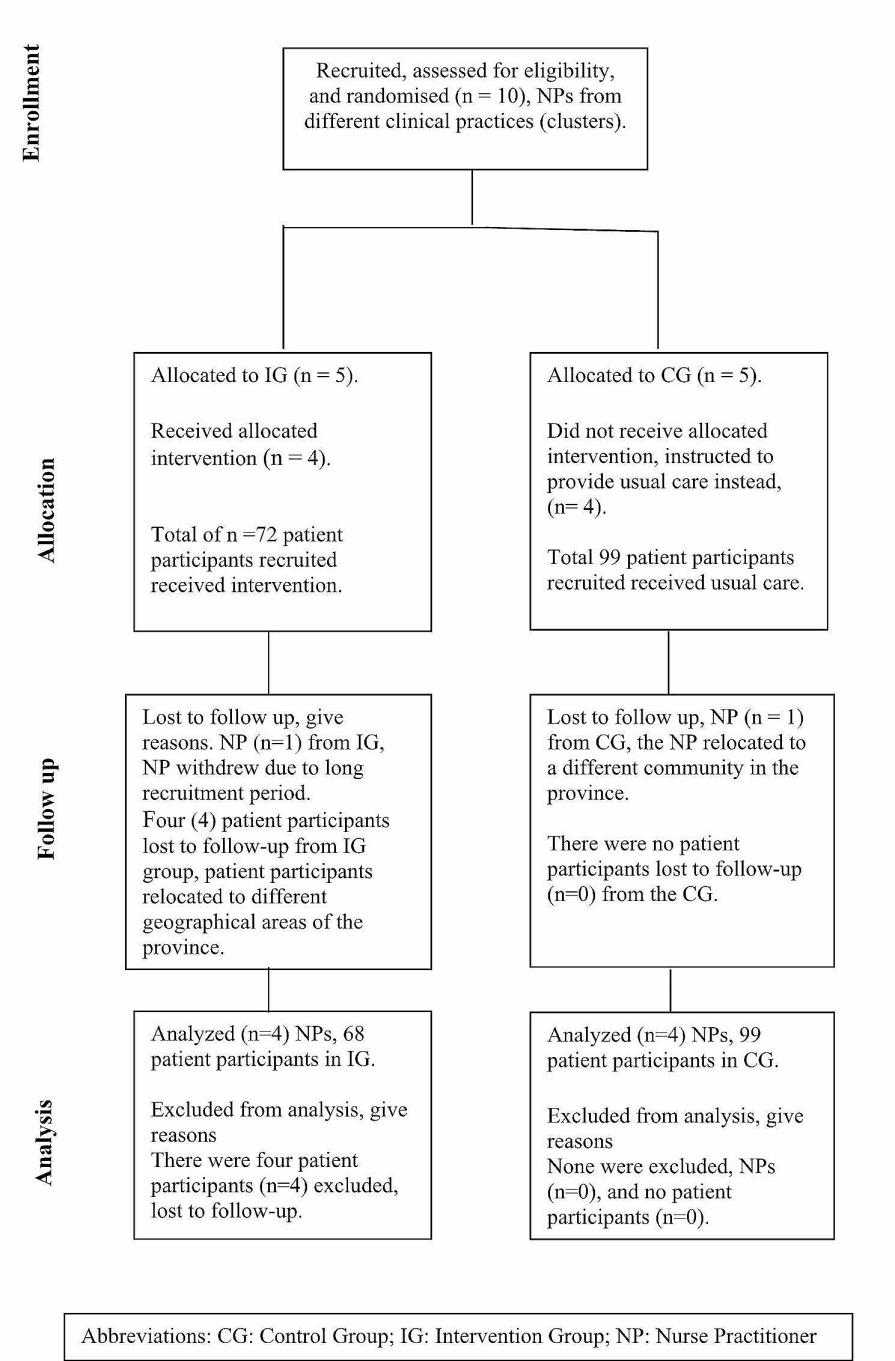
Flow of NPs and patient participants from recruitment to analysis

The participant flow diagram for this study shows the flow of NPs recruited as well as the flow of patient participants and indicates the number of NPs randomly assigned, patients that received intended treatment, and patients analysed for the primary outcome, see Fig. 1. For each group, losses and exclusions for both NPs and individual patient participants are shown from recruitment to analysis. Researchers limited the data analysis to only include the participant data that was complete; missing data were treated as not done and were omitted.

Recruitment of NPs occurred between September 2017 and March 2018. Following random assignment, the NPs successfully recruited and followed up with individual patient participants from March 2018 to the end of the trial period in November 2018.

### Baseline data characteristics

#### NPs

A total of eight NPs participated in the study. The NPs in both IG and CG groups were comparable in age, with most NPs over 45 years. Only one NP in the IG was in a younger age category 25–34 years. Both IG and CG were similar in sex, with each group having three females and one male NP. In IG, three of the NPs had less than 10 years working as NPs compared to those NPs in the CG who all had over 10 years of experience. Overall, the NP baseline characteristics were comparable, but varied by years of working experience, which was controlled for in the statistical analysis.

#### Patients

As shown in Table 1, baseline characteristics of individual patient participants in the IG and CG were similar with the exception of the distribution of patients across NL, with no participants in the CG from two RHAs. Participants in the IG were similar to those in the CG in terms of education, age, and gender. Table 1 also shows the results of patients’ documented comorbidities for the IG compared to the CG. Compared to the CG, a significantly (*p* < .05) higher proportion of patients in the IG had abnormal blood pressure, elevated blood glucose, and renal dysfunction, while the presence of dyslipidemia was similar for both groups. However, comorbidities were unknown in 21–66% of the patients in the CG because of lack of documentation in their charts, compared to fewer than 10% of patients in the IG.

**Table 1:** Baseline characteristics of patient participants

Baseline characteristics		Intervention group^1^	Control group^1^
**Regional** **Health Authority** ***p*** **< .001**	Eastern	9 (13.2%)	69 (69.7%)
	Central	11 (16.2%)	30 (30.3%)
	Western	30 (44.1%)	0 (0%)
	Labrador-Grenfell	18 (26.5%)	0 (0%)
**Education**	Less than high school	19 (27.9%)	6 (6.1%)
	High school	30 (44.1%)	5 (5.1%)
	Undergraduate	14 (20.6%)	2 (2.0%)
	Graduate degree	5 (7.4%)	0 (0%)
***p*** = .501*	Unknown	0 (0%)	86 (86.9%)
**Age** ***p*** **= .793**	Mean	55 years	56 years
	Range	40–74 years	40–74 years
**Gender** ***p*** **= .633**	Males	18 (25%)	23(23.2%)
	Females	50 (75%)	76 (76.8%)
**Blood pressure** ^**2**^ ***p*** = .104*	Normal	43 (63%)	51 (52%)
	Abnormal	25 (38%)	16 (16%)
	Unknown	0 (0%)	32 (32%)
**Diabetes** ^**3**^ ***p*** = .616*	Normal	44 (65%)	45 (45.4%)
	Abnormal	18 (26%)	15 (15.1%)
	Unknown	6 (9%)	39 (39.3%)
**Lipid Profile** ^**4**^ ***p*** = .953*	Normal	27 (27%)	24 (35%)
	Abnormal	33 (33%)	30 (30%)
	Unknown	8 (8%)	45 (45%)
**Renal Function** ^**5**^ ***p*** = .015*	Normal	40 (59%)	29 (29%)
	Abnormal	22 (15%)	4 (4%)
	Unknown	6 (9%)	66 (66%)

^1^N (%): the number and percentage of patients in each group with the identified characteristic; there were 68 patients in the intervention group and 99 patients in the control group. ^2^Blood Pressure: Normal: BP ≤ 140/90; abnormal BP > 140/90 ^3^Diabetes: Normal: A1C ≤ 7; abnormal: A1C > 7 ^4^ Lipid Profile: documented as abnormal if levels of any of the following were elevated: Total cholesterol, Total cholesterol/HDL: ratio, LDL, triglycerides, or total cholesterol. ^5^Renal Function: Normal albumin-creatinine ratio: ACR < 1.8 or eGFR > 60; abnormal: ACR > 1.8 or eGFR < 60) *p values calculated for differences between groups for participants with documented data (e.g., documented education level or A1C). P values were calculated using chi square for categorical variables and independent t-test for the continuous variable (age)

NPs in eight different community-based practices participated in this study with 167 individual patient participants included in the analysis. The data were analysed by the originally assigned groups (IG and CG).

#### Primary outcome

The primary outcome of this cRCT was comprehensiveness of CVD screening. The IG NPs utilised all of the CASP components in order to screen comprehensively. As shown in Table 2, the majority of patients in the IG had comprehensive screening compared to the CG. There was a statistically significant difference between IG NPs using CASP and doing comprehensive screening (identifying at least 9 of the 10 specified risk components) compared to CG NPs providing usual care. In the GLM, researchers assessed the following as potential confounders or significant predictors: age, gender, BP category, diabetes, smoking status, renal dysfunction, and RHA. In addition to including the NP to control for the cluster effect, the final model only contained BP category as a confounder. According to the final model, more patients received comprehensive screening in the IG (90%; *n* = 61) versus the CG (2%; *n* = 2), RR = 30.2, 95% CI [8.76, 103.9], *p* < .0001, when the effect of NP and BP category were controlled for. The participants in the IG were much more likely (30 times) to have comprehensive screening compared to the participants in CG. The CI was wide, but even the lower limit of 8.76 indicates a significant effect on how the NPs in the IG used a comprehensive approach to screening when implementing the CASP intervention, when controlling for the effect of the NP.

**Table 2:** Degree of comprehensive screening comparison between participants in the intervention and control groups

Degree of comprehensive CVD screening^1^	Intervention group^2^	Control group^2^
Comprehensive CVD screening^3^ (9–10 components)	**90% (61)**	2% (2)
Moderate CVD screening^4^ (6–8 components)	**10% (7)**	1% (1)
Limited CVD screening^5^ (3–5 components)	0%(0)	**54% (54)**
Minimal CVD screening^6^ (1–2 components)	0% (0)	**42% (42)**

^1^There was a significant difference between the intervention and control group for the level of screening *p* < .0001. ^2 ^% (N): the percentage and number of patients in each group with the identified characteristic; there were 68 patients in the intervention group and 99 patients in the control group. ^3^ Comprehensive CVD screening was based on the NPs obtaining information from the patients on 9 or 10 of the following components: age, family history of premature coronary artery disease, Framingham Risk Score, smoking status, body mass index, waist circumference, blood pressure, lipid profile, A1C, and stress. Screening was categorized as ^4^moderate if 6–8 components were evaluated, as ^5^limited if 3–5 components were evaluated and ^6^minimal if 1–2 components were evaluated

### Secondary outcomes

There were three main secondary outcomes in the cRCT: (1) the identification of risk factors, (2) the level of CVD risk measured by calculating the FRS and Heart Age, and (3) the identification of priorities for heart health and setting personalised goals. The NPs asked the patients about their priorities for heart health and this information was documented in the CVD database for later analysis.

#### Identification of risk factors

As shown in Table 3, patients had more risk factors documented by NPs in the IG compared to the CG. The majority (71%) of IG participants had four or more risk factors for CVD documented including premature family history of CAD, smoking, hypertension, diabetes, obesity, renal dysfunction, and dyslipidemia compared to the only 5% of CG participants. Documented risk factors included premature family history of CVD, smoking, hypertension, diabetes, obesity, renal dysfunction, and dyslipidemia.

**Table 3:** CVD risk factors of participants in the intervention and control groups

Number of risk factors	IG participants^1^	Sex^2^		CG participants^3^	Sex^4^	
7–10	12 (18%)	Female	7 (14%)	0 (0%)	Female	0 (0%)
		Male	5 (27%)		Male	0 (0%)
4–6	36 (53%)	Female	28 (56%)	5 (5%)	Female	3 (4%)
		Male	8 (44%)		Male	2 (8%)
2–3	16 (23%)	Female	14 (28%)	46 (46%)	Female	35 (46%)
		Male	2 (11%)		Male	11 (48%)
0–1	2 (3%)	Female	1 (2%)	22 (22%)	Female	16 (21%)
		Male	1 (5%)		Male	6 (26%)
Unknown	2 (3%)	Female	0 (0%)	26 (26%)	Female	22 (29%)
		Male	2 (11%)		Male	4 (17%)

^1^ N (%) the number and percentage of participants in the IG (intervention group); there were 68. ^2^ N (%) the number and percentage of participants in the IG who had the given number of risk factors assessed by sex; there were 50 females and 18 males. There was no significant difference by sex, *p* = .299. ^3^N (%) the number and percentage of patients in the CG (control group); there were 99 participants. ^4^N (%) the number and percentage of participants in the CG who had the given number of risk factors assessed by sex; there were 76 females and 23 males. There was no significant difference by sex, p = .070

In the IG, a higher proportion of males (27%) than females (14%) had 7–10 risk factors components assessed, while higher proportions of females (28–56%) than males (11–44%) had 2–6 components assessed. In the CG, a slightly higher proportion of males than females had components assessed, for the categories of 4–6, 2–3 and 0–1 components.

#### Level of CVD risk

The NPs in the IG assessed and documented the results of the FRS for 91% (*n* = 62) of the IG patients. In comparison, the risk for having a CV event was largely unknown for 96% (92 participants) of the control group because the FRS was documented on only seven (7) patient participants (4%). The difference was statistically significant (*p* < .001). NPs in the IG also calculated and communicated the Heart Age with their patients during the implementation of the CASP intervention. In the IG, 91% (*n* = 62) of participants had their Heart Age documented but the NPs in the CG did not document Heart Age for the participants at all.

#### Identification of priorities for heart health and setting personalised goals

As part of the CASP intervention, NPs in the IG identified and documented priorities for patient management based on their CVD screening and identified management strategies using current CPGs embedded in the CASP components. They used a variety of CASP resources when counselling patients. Determining priorities was defined as identifying specific risk factors to be addressed in order to improve heart health. Priorities or recommendations by NPs in the IG for their patients were the following: reducing salt intake, losing weight, controlling glucose level, or increasing physical exercise. Each patient also identified what their heart health priorities were following the clinic visit with the NP who communicated with them about their unique risk factors and conditions. The vast majority (92%) of patients had priorities identified by each NP in the IG. There was variation in the number of patient priorities identified; however, all NPs in the IG identified two to three patient priorities for at least 75% of the participants. Ninety-four percent (94%) of the priorities for heart health identified by the NP in the IG were the same as the priorities identified by the patient participants. Over three quarters (80%) of the patient participants in the IG identified two or more priorities for improving heart health. It was not possible to compare the priorities set by the NPs in the CG because the NPs in CG did not clearly document in the charts regarding their priorities or the priorities of CG participants related to CVD screening and management.

## Discussion

In the cRCT, there was a significant difference in comprehensive screening with the NPs in the IG implementing and testing the CASP intervention in community practice settings compared to the NPs providing usual care in the CG. The NPs in the IG evaluating CASP successfully performed comprehensive CVD screening, identified risk factors, communicated level of CVD risk, determined priorities for heart health, and helped patients set personalised goals.

The novel CASP intervention, utilised by NPs in this study as a clinical tool, operationalised the C-CHANGE Guideline to promote comprehensive, systematic CVD screening in daily practice. Comprehensive screening meant that NPs were able to gain in-depth knowledge about their patients’ heart health status from physiological findings, critical blood work results, risk calculations, and insight into the individuals’ priorities for heart health within a short time period. Then, NPs were able to engage, communicate, and collaborate with their patients so to assist patients to develop their priorities for heart health and personalised goals. Because there may be differences in the screening practices of the NPs (e.g., due to their knowledge or practice settings), we used GLM to control for the effect of the NP and still found a significant effect of CASP on comprehensiveness of screening. Interestingly, there are few national screening programs for comparison, and they do not focus on comprehensiveness of screening as a measure of success. The UK program, for example, measures success in risk factor assessment by the proportion of the population who are participating in the NHS Health Check Program or the uptake of the program in different regions [18].

The CASP intervention guided the NPs in the IG to the specific risk factors to assess, and how to screen for them, as well as facilitated documentation of both what was screened for and what was found. There were many risk factors identified and this certainly justifies the early screening, otherwise these risks for CVD are missed potentially leading to premature morbidity and mortality. It is not surprising that implementing CASP resulted in the effective identification of risk factors since other screening programs have been shown to be effective in identifying risk factors, such as hypertension, type 2 diabetes, chronic kidney disease [19–22].

Many HCPs, in the past, thought they were the “experts” of health knowledge and were in control of determining priorities and responsible for the actions of the patients in their care. This approach is both inappropriate and ineffective in changing behavior [23]. At present, focusing on patient-centred care and shared decision-making rather than provider-driven priorities is critically important. In addition, evidence that using motivational interviewing in patient-centred approaches can enhance behaviour change in many individuals [24, 25]. Discussion and sharing of priorities and goals and the use of motivational interviewing therefore were important aspects of the CASP intervention. Investigating the congruence between priorities for action following communication of risk assessment results and focusing on patient-centered goals related to heart health, has not, to our knowledge, been previously studied. A future study can evaluate the effectiveness of this shared priority setting on patient behaviours and outcomes.

Because of the missing data in the charts of the patients in the CG, and the limited or minimal documentation of screening activities on the CG patients, it was unclear what their actual risks were for CVD or if the patients were aware of their risks. However, we do not think that the effect of CASP was greatly overestimated because physical findings relevant to the CVD screening (e.g., waist circumference), specific lab tests, or FRS, were not documented in CG charts and would have been documented if they had been done. As a result, because of this missing data in the CG, direct comparisons with the IG were not possible. There was minimal patient data missing from the IG, so this missing data was treated as unknown. As well, there were two regions of the province not represented in the CG, therefore, no data from these areas were included in the analyses which may limit generalisability to these regions. There were challenges in recruiting NPs for this study as they are busy HCPs with limited organizational support for participating in research [13].

In addition to promoting comprehensive screening CASP can help facilitate access to screening and appropriate care. Access to primary health care is limited in the province of NL and in many parts of rural Canada, due to sparsely populated large geographical areas in rural and remote communities. To improve access to care, NPs living in rural areas of NL were able to implement the CASP intervention during this study mostly because the components of this intervention were online such as the CASP website, CVD database, etc. Improving access to screening earlier in the lifespan in both urban and rural areas through the implementation of CASP utilising virtual care, ensured identification of risk factors and conditions in a timely manner impacting management of patients with current treatments and recommendations according to current evidence.

Knowledge translation of evidence into practice is challenging for HCPs such as NPs and physicians working in community and other health care settings. NPs are ideally positioned within the healthcare system to identify risk factors, order specific diagnostic tests, prescribe current therapies, refer patients to other team members, and engage in individualised counseling to contribute to the reduction of CVD morbidity and improve health outcomes. NPs work in both urban and rural settings and they are often the only providers in very remote areas. Patients in these remote rural areas may otherwise have difficulty accessing appropriate CVD risk factor assessment and appropriate management using current guidelines.

In addition to the limited documentation in the CG charts, as previously discussed, limitations of the study related to the small sample size, choice of risk factors for screening, the short duration of the study, and generalisability. A small sample size of NPs in the clusters and patient participants limited the ability to use more variables than we did in the regression to control for potential confounders. Researchers selected specific risk factors and conditions from the C-CHANGE guideline for NPs to focus on collecting and for inclusion in the CVD database. The question remains about whether we focused on the correct risk factors, measurements, and risk calculators for screening comprehensively. There are other CVD risk factors that may be considered more important to use that were not included, but could be assessed in a future study. The short duration of the study precluded assessing the impact of the intervention on patient behavior and outcomes. Due to the small sample size in one Canadian province generalisability of results are limited.

## Conclusions

The focus of the study was on evaluating CASP as an intervention to strengthen screening for CVD risk factors, using a systematic and comprehensive approach, rather than determining prevalence of risk factors. A key finding was that comprehensive screening was significantly higher in the IG compared to the CG (RR = 30.2, 95% CI [8.76, 103.9], *p* < .0001). Because screening was more comprehensive, we also found that patients in the IG had more risk factors documented than the CG patients, including the FRS and Heart Age. They also had clear heart health-related priorities and personalised goals documented. Increased screening and identification of risk factors will enable patients and NPs to take action to address the risk factors, thus potentially improving heart health. The implications for practice and research are described below.

- Our evidence-informed CASP intervention was successful in promoting comprehensive CVD screening and can be another clinical tool for NPs and other practitioners to use in daily practice.
- Patient engagement achieved during this cRCT provides an excellent example of patient-centred care and HCPs utilising similar methods can enhance care provided in daily patient encounters.
- Because the CASP intervention used an online format, increased accessibility to appropriate care by NPs was possible with at-risk individuals living in both rural and remote communities.
- NPs were able to engage patients to participate in CVD screening, identify multiple risk factors, follow up with patients to communicate screening results, and collaborate with patients to develop personalised goals for heart health. Other HCPs can perform similar actions and collaborate with their patients to reduce CVD risk.
- Future research on the implementation of CASP by NPs and other HCPs could enhance the uptake of the C-CHANGE guideline, or other current evidence commonly used in clinical practice, to potentially reduce CVD risk of populations in the future.

## Data Availability

The data that support the findings of this study are available from Memorial University of Newfoundland (MUN), but restrictions apply to the availability of these data, so are not publicly available. The data are, however, available from the authors upon reasonable request and with the permission of MUN Research Ethics Board (HREB).

## Declarations

### Registration

ClinicalTrials.gov (Identifier: NCT03170752) registry

## Abbreviations

A1C: Glycated Hemoglobin
BP: Blood Pressure
BMI: Body Mass Index
CG: Control Group
CAD: Coronary Artery Disease
CASP: Cardiovascular Assessment Screening Program
C-CHANGE: Canadian Cardiovascular Harmonized National Guideline Endeavour
CPG: Clinical Practice Guideline
CVD: Cardiovascular Disease
FRS: Framingham Risk Score
cRCT: Cluster Randomised Controlled Trial
HCP: Healthcare Provider
HREB: Health Research Ethics Board
IG: Intervention Group
NHS: National Health Service
NL: Newfoundland and Labrador
NP: Nurse Practitioner
PI: Principal Investigator
PHAC: Public Health Agency of Canada
RHAs: Regional Health Authorities
RPACs: Research Proposal Approval Committees
WC: Waist Circumference
WHO: World Health Organization

## References

[1] World Health Organization Cardiovascular Diseases. Key Facts. Retrieved from WHO May 15 2022: https://www.who.int/news-room/fact-sheets/detail/cardiovascular-diseases-(cvds)#:~:text=Cardiovascular%20diseases%20(CVDs)%20are%20the,%2D%20and%20middle%2Dincome%20countries.

[2] Public Health Agency of Canada. (2017). Heart Disease in Canada: Highlights from the Canadian Chronic Disease Surveillance System. Retrieved on February 18, 2019 from: https://www.canada.ca/en/public-health/services/publications/diseases-conditions/heart-disease-canada-fact-sheet.html.

[3] Heart and Stroke Foundation. 2019. Retrieved from: www.heartandstroke.ca.

[4] World Health Organization. (2017). Fact Sheet Cardiovascular Diseases (CVDs). Retrieved from: https://www.who.int/news-room/fact-sheets/detail/cardiovascular-diseases-(cvds).

[5] Alvarez-Bueno C, Cavero-Redondo I, Martinez-Andres M, Arias-Palencia N, Ramos-Blanes R, Salcedo-Aguilar F (2015). Effectiveness of multifactorial interventions in primary health care settings for primary prevention of cardiovascular disease: a systematic review of systematic reviews. Prev Med.

[6] Tobe SW, Stone JA, Anderson T, Bacon S, Cheng AYY, Daskalopoulou SS (2018). Canadian Cardiovascular Harmonized National guidelines Endeavour (C-CHANGE) guideline for the prevention and management of cardiovascular disease in primary care: 2018 update. CMAJ.

[7] Peters S, Sukumar K, Blanchard S, Ramasamy A, Malinowski J, Ginex P (2022). Trends in guideline implementation: an updated scoping review. Implement Sci.

[8] Dyakova M, Shantikumar S, Colquitt JL, Drew CM, Sime M, MacIver J (2019). Systematic versus opportunistic risk assessment for the primary prevention of cardiovascular disease. Cochrane Database Syst Rev.

[9] Unverzagt S, Oemler M, Braun K, Klement A (2014). Strategies for guideline implementation in primary care focusing on patients with cardiovascular disease: systematic review. Fam Pract.

[10] Bruneau J, Parsons K, Moralejo D, Donovan C. Development of the Cardiovascular Assessment Screening Program (CASP) using the qualitative findings of a mixed methods study and applying the TDF to address the barriers of and facilitators to comprehensive screening for cardiovascular disease. BMC Prim Care. 2023 March;24(1):1–6. 10.1186/s12875-023-02022-8 PMC9990229 36882713

[11] Bruneau J, Moralejo D, Donovan C, Parsons K (2020). The development and evaluation of the cardiovascular assessment screening program. Meml Univ Res Repository.

[12] Campbell MK, Piaggio G, Elbourne DR, Altman DG. Consort 2010 statement: extension to cluster randomised trials. BMJ. 2012;345. 10.1136/bmj.e5661 22951546

[13] Bruneau J, Moralejo D, Donovan C, Parsons K (2021). Recruitment of Healthcare providers into Research studies. Can J Nurs Res.

[14] Centre for Collaboration. Motivation and Innovation. Retrieved from: www.centrecmi.ca.

[15] Artac M, Dalton AR, Majeed A, Car J, Millett C (2013). Effectiveness of a national cardiovascular disease risk assessment program (NHS health check): results after one year. Prev Med.

[16] STATA 17. STATA software for statistics and data science. 2022 Retrieved from: www.stata.com.

[17] STATA 13. STATA software for statistics and data science. 2013. Retrieved from: www.stata.com.

[18] Kennedy O, Su F, Pears R, Walmsley E, Roderick P (2019). Evaluating the effectiveness of the NHS Health Check programme in South England: a quasi-randomised controlled trial. BMJ open.

[19] Eapen ZJ, Liang L, Shubrook JH, Bauman MA, Bufalino VJ, Bhatt DL (2014). Current quality of cardiovascular prevention for million hearts: an analysis of 147,038 outpatients from the Guideline advantage. Am Heart J.

[20] Kelsall HL, Fernando PHS, Gwini SM, Sim MR (2018). Cardiovascular disease and type 2 diabetes risk across occupational groups and industry in a statewide study of an Australian working population. J Occup Environ Med.

[21] Lindholt JS, Rasmussen LM, Søgaard R, Lambrechtsen J, Steffensen FH, Frost L (2019). Baseline findings of the population-based, randomized, multifaceted Danish cardiovascular screening trial (DANCAVAS) of men aged 65–74 years. J Br Surg.

[22] Alageel S, Gulliford MC. Health checks and cardiovascular risk factor values over six years’ follow-up: Matched cohort study using electronic health records in England. PLoS Med, 2019;16(7), e1002863. 10.1371/journal.pmed.1002863 PMC6667114 31361740

[23] Rollnick S, Miller WR, Butler CC. Motivational interviewing in health care helping patients change behaviour. The Guilford; 2008.

[24] Waldron CA, van der Weijden T, Ludt S, Gallacher J, Elwyn G (2011). What are effective strategies to communicate cardiovascular risk information to patients? A systematic review. Patient Educ Couns.

[25] Lundhal B, Droubay BA, Burke B, Butters RP, Nelford K, Hardy C et al. Motivational interviewing adherence tools: a scoping review investigating content validity. Patient Educ Couns 2019; Dec. 102(12), 2145–55. 10.1016/j.pec.2019.07.003 31514978

